# ﻿Three new species of *Apiospora* (Apiosporaceae, Amphisphaeriales) associated with diseased bamboo in China

**DOI:** 10.3897/mycokeys.116.142263

**Published:** 2025-04-23

**Authors:** Xiaoyun Chang, Yuanyuan Wang, Tao Xu, Guangshuo Li, Xianghua Yue, MingJun Chen

**Affiliations:** 1 Anhui Province Key Laboratory of Green Control for Major Forestry Pests, Anhui Agricultural University, Hefei 230036, China; 2 Key Laboratory of Biology and Sustainable Management of Plant Diseases and Pests of Anhui Higher Education Institutes, School of Plant Protection, Anhui Agricultural University, Hefei 230036, China; 3 International Centre for Bamboo and Rattan, Sanya Research Base, Sanya 572000, China

**Keywords:** Bambusicolous fungi, morphology, new taxa, phylogeny, taxonomy

## Abstract

*Apiospora* is widely distributed worldwide, primarily comprising pathogens, endophytes, and saprobes associated with plants, and most of its hosts are Poaceae. In this study, 37 pathogenic strains of *Apiospora* were isolated from diseased bamboo collected in the provinces of Hunan and Guizhou, China. Multilocus phylogenetic analysis using combined ITS, LSU, *TUB2*, and *TEF1* sequence data, along with morphological assessments, identified three new species: *A.bambusiparasitica***sp. nov.**, *A.qiannanensis***sp. nov.**, and *A.xiangxiense***sp. nov.** Descriptions, illustrations, and phylogenetic trees for the newly discovered species are provided and compared with closely related *Apiospora* species to enhance our understanding of the genus *Apiospora*. The pathogenicity test results demonstrated that the three new species could cause bamboo culm diseases, providing valuable information for the diagnosis and management of bamboo culm diseases.

## ﻿Introduction

The genus *Apiospora* (Amphisphaeriales, Apiosporaceae) was established and described by Saccardo in 1875, with *Apiosporamontagnei* (Saccardo 1875) designated as the type species. The ongoing development of fungal taxonomy and phylogeny has led to multiple revisions of the taxonomic status of the genus *Apiospora*. Before 2021, the phylogenetic relationship between *Arthrinium* and *Apiospora* remained unclear. Molecular phylogenetic studies initially placed both genera in the family Apiosporaceae (Hyde et al. 1998). Subsequently, Crous and Groenewald (2013) proposed synonymizing *Arthrinium* with *Apiospora* and prioritized the former as per the “one fungus, one name” policy (Hawksworth et al. 2011; Réblová et al. 2016), despite a lack of data on the type species *Arthriniumcaricicola*. However, Pintos and Alvarado’s (2021) study showed that genetic, morphological, and ecological differences between *Apiospora* and *Arthrinium* were considered sufficient to support the taxonomic separation of the two genera. Furthermore, Pintos and Alvarado (2022) refined the identity of *Apiosporamontagnei* as the type species and delineated its phylogenetic boundaries. Additionally, many species from *Arthrinium* were transferred to *Apiospora* (Pintos and Alvarado 2021; Tian et al. 2021). As of March 2025, there are 206 epithets listed in Index Fungorum.

Morphologically, *Apiospora* and *Arthrinium* share many similarities, particularly in their asexual characteristics (Yuan et al. 2020). However, most *Apiospora* conidia are nearly spherical from the front view and lenticular from the side, while *Arthrinium* often produces conidia of various shapes, including angular, spherical, curved, boat-shaped, fusiform, and polygonal (Crous and Groenewald 2013; Pintos and Alvarado 2021, 2022; Li et al. 2023; Ai et al. 2024; Liu et al. 2024).

Ecologically, *Apiospora* is mainly associated with Poaceae or other plant hosts in tropical and subtropical regions (Liao et al. 2023; Liu et al. 2023; Zhang et al. 2023), while *Arthrinium* primarily occurs on Cyperaceae or Juncaceae hosts in temperate, cold, or alpine habitats (Sharma et al. 2014; Kwon et al. 2021; Pintos and Alvarado 2022). Current fungal taxonomy also emphasizes the correlation between host specificity and geographic location. All these pieces of evidence support the taxonomic separation of the two genera.

Bamboo is a vital non-wood bioresource, playing an irreplaceable role in economic, ecological, medicinal, and societal development (Shukla et al. 2016; Borowski et al. 2022). However, the intensification of bamboo cultivation has heightened its vulnerability to infectious diseases. Among fungal pathogens associated with bamboo, obligate pathogens such as *Phyllachora*, *Physopella*, *Puccinia*, *Stereostratum*, and *Uredopredominantly* infect bamboo leaves, while sporadic pathogens, including *Apiospora*, *Meliola*, *Fusarium*, and *Sclerotium*, target both leaves and culms (Hyde et al. 2002; Yang et al. 2019). The pathogenicity of *Apiospora* species on bamboo has garnered increasing attention. For instance, Li et al. (2016) identified *A.phaeospermum* as the causative agent of culm rot in *Phyllostachysviridis* in China. Subsequently, Yang et al. (2019) reported *A.yunnanum* as the pathogen responsible for bamboo blight in *Phyllostachysheteroclada*. More recently, Zheng et al. (2022) confirmed that *A.arundinis* caused culm rhomboid rot in Moso bamboo (*Phyllostachysedulis*). Beyond their pathogenic roles, several *Apiospora* species are recognized as endophytes, contributing to the microbial diversity of bamboo (Wang et al. 2018).

In this study, we isolated several *Arthrinium*-like taxa from diseased culms of bamboo in China. To clarify their taxonomic status, we used a dataset composed of nuclear ribosomal DNA internal transcribed spacer (ITS), large subunit ribosomal DNA (LSU), β-tubulin (*TUB2*), and translation elongation factor 1-α (*TEF1*). Based on morphological characteristics and multi-gene phylogenetic analyses, we identified and described three new *Apiospora* species.

## ﻿Materials and methods

### ﻿Plant material

In this study, diseased bamboo samples were collected from Jiuyi Mountain in Ningyuan County, Xiangxi Tujia and Miao Autonomous Prefecture, Hunan, and from Libo County, Qiannan Buyi and Miao Autonomous Prefecture, Guizhou, China. The International Center for Bamboo and Rattan provided the specimens. Samples were deposited in the Research Center for Entomogenous Fungi (RCEF) of Anhui Agricultural University.

### ﻿Pathogen isolation

Pure cultures of all fungal isolates were obtained by the single hyphal tip isolation method. For pathogen isolation, lesion margin specimens were excised into 5 × 5 mm fragments, surface-sterilized in 2% sodium hypochlorite for 2 min, followed by immersion in 75% ethanol for 1 min, and rinsed three times consecutively with sterile water (Zheng et al. 2022). The sterilized pieces were wiped dry with sterilized filter paper and then placed into Petri dishes containing potato dextrose agar (PDA) (three pieces per dish) amended with 50 µg/mL of benzylpenicillin potassium (Cai et al. 2009). The plates were incubated at 25 °C under a 12 h light/dark photoperiod. Hyphal tips from the leading edge of fungal colonies emerging from the tissues were transferred to fresh PDA after two days to obtain pure cultures, which were subsequently maintained at 25 °C. Living cultures were stored in a metabolically inactive state at the Research Center for Entomogenous Fungi (RCEF) of Anhui Agricultural University. The MycoBank number for the newly described species is referenced as outlined in Robert et al. (2013).

### ﻿Morphological characterization

For morphological identification, the purified isolated strains were incubated on PDA (fresh diced potato 200 g/L, dextrose 20 g/L, agar 20 g/L) and MEA (malt extract 20 g/L and 20 g/L agar) at 25 °C. Incubate at 25 °C in alternating light and dark (12 h for each); colony growth was observed daily, and the morphology, color, texture of colonies, and the diameter of colonies were recorded. Asexual reproductive structures were observed based on cultures on PDA, following synoptic keys for *Apiospora* species identification. In the morphological analysis, the fungi were mounted in a drop of lactophenol solution on glass slides. The microstructures, such as mycelium, conidiogenous cells, and conidia, were observed using an optical microscope (ZEISS Axiolab 5) and microphotographed. Forty conidiogenous cells and conidia were measured and examined. The colors of fresh specimens and cultures were recorded by referring to the Methuen Handbook of Color (Kornerup and Wanscher 1978).

### ﻿DNA extraction and PCR amplification

The genomic DNA of the isolates was extracted from mycelium that was cultured on a PDA plate and incubated for 3–5 days at 25 °C. DNA extraction was performed according to the CTAB method (Spatafora et al. 1998).

Polymerase chain reaction (PCR) amplification was applied to amplify four gene fragments, including ITS, LSU, *TUB2*, and *TEF1*. The following primer pairs were used: ITS1/ITS4 for ITS (White et al. 1990), LR0R/LR5 for LSU (Rehner and Samuels 1995), EF1-728F/EF2 for *TEF1* (O’Donnell et al. 1998; Carbone and Kohn 1999), and T1/Bt2b for *TUB2* (Glass and Donaldson 1995; O’Donnell and Cigelnik 1997). The PCR amplification system consisted of 2 min at 94 °C, followed by 35 cycles of 30 s at 94 °C, 30 s at 48 °C (ITS), and 45 s at 72 °C, and a final step of 2 min at 72 °C. Different annealing temperatures were used according to the genomic region to be amplified: 56 °C for *TEF1* and LSU, 58 °C for *TUB2*. The final product was detected by agarose gel electrophoresis and sent to Beijing Liuhe Huada Gene Technology Company. The resulting sequences were submitted to GenBank for sequencing.

### ﻿Molecular and phylogenetic analysis

Newly generated sequences from each isolate were blasted against the GenBank database, and searches were restricted to type materials for the initial determination of the closest matching species and species complex. Related gene sequences (ITS, LSU, *TUB2*, *TEF1*) of *Apiospora* spp. from recent publications were downloaded from GenBank (Table 1) (Yan and Zhang 2024). Manual adjustments of sequences were carried out using BioEdit (Hall et al. 2011) to maximize homology.

**Table 1.** Species of Apiosporaceae used in the phylogenetic analyses. Notes: Strains in this study are marked in bold. “T” indicates a type culture. NA = not available.

Strain	Code	Host and Substrates	Locality	GenBank accession numbers			
				ITS	LSU	*TUB2*	*TEF1*
*Apiosporaacutiapica*	KUMCC 20-0209	*Bambusabambos*	China	MT946342	MT946338	MT947365	MT947359
*A.adinandrae*	SAUCC 1282B-1 ^T^	Diseased leaves of *Adinandraglischroloma*	China	OR739431	OR739572	OR757128	OR753448
*A.agari*	KUC21333 ^T^	*Agarumcribrosum*	South Korea	MH498520	MH498440	MH498478	MH544663
*A.aquatica*	S-642 ^T^	Submerged wood	China	MK828608	MK835806	NA	NA
*A.arctoscopi*	KUC21331 ^T^	Eggs of *Arctoscopusjaponicus*	South Korea	MH498529	MH498449	MH498487	MN868918
*A.armeniaca*	SAUCC DL1831 ^T^	Leaves of *Prunusarmeniaca*	China	OQ592540	OQ615269	OQ613285	OQ613313
*A.arundinis*	CBS 124788	Living leaves of *Fagussylvatica*	Switzerland	KF144885	KF144929	KF144975	KF145017
*A.aseptata*	KUNCC 23-14169 ^T^	Living roots of *Dicranopterispedata*	China	OR590341	OR590335	OR634943	OR634949
*A.aurea*	CBS 244.83 ^T^	Air	Spain	AB220251	KF144935	KF144981	KF145023
*A.babylonica*	SAUCC DL1841 ^T^	Diseased leaves of *Salixbabylonica*	China	OQ592538	OQ615267	OQ613283	OQ613311
*A.balearica*	AP24118 ^T^	Poaceae plant	Spain	MK014869	MK014836	MK017975	MK017946
*A.bambusicola*	MFLUCC 20-0144 ^T^	*Schizostachyumbrachycladum*	Thailand	MW173030	MW173087	NA	MW183262
***A.bambusiparasitica***	**RCEF20000**	**Diseased culms of bamboo**	**China**	**OR687309**	**PQ530552**	**OR712912**	**PQ538537**
***A.bambusiparasitica***	**RCEF20003** ^T^	**Diseased culms of bamboo**	**China**	**OR687306**	**PQ530551**	**OR712906**	**OR712911**
*A.bawanglingensis*	SAUCC BW0444 ^T^	Leaves of *Indocalamuslongiauritus*	China	OR739429	OR739570	OR757126	OR753446
*A.bawanglingensis*	SAUCC 0443	Diseased leaves of *Indocalamuslongiauritus*	China	OQ592552	OQ615281	OQ613303	OQ613325
*A.bawanglingensis*	SAUCC 0444	Diseased leaves of *Indocalamuslongiauritus*	China	OQ592551	OQ615280	OQ613302	OQ613324
*A.biserialis*	CGMCC 3.20135 ^T^	Bamboo	China	MW481708	MW478885	MW522955	MW522938
*A.camelliae-sinensis*	LC5007 ^T^	*Camelliasinensis*	China	KY494704	KY494780	KY705173	KY705103
*A.cannae*	ZHKUCC 22-0139	Leaves of *Canna* sp.	China	OR164902	OR164949	OR166322	OR166286
*A.chiangraiense*	MFLU 21-0046	Dead culms of bamboo	Thailand	MZ542520	MZ542524	MZ546409	NA
*A.chromolaenae*	MFLUCC 17-1505 ^T^	*Chromolaenaodorata*	Thailand	MT214342	MT214436	NA	MT235802
*A.cordylines*	GUCC 10026	*Cordylinefruticosa*	China	MT040105	NA	MT040147	MT040126
*A.coryli*	CFCC 58978 ^T^	Dead plant culms of *Corylusyunnanensis*	China	OR125564	OR133586	OR139978	OR139974
*A.cyclobalanopsidis*	GZCC 20-0103	*Cyclobalanopsidisglauca*	China	MW481714	MW478893	MW522963	MW522946
*A.dematiacea*	KUNCC 23-14202 ^T^	Healthy leaf *Dicranopterisampla*	China	OR590346	OR590339	OR634948	OR634953
*A.dendrobii*	MFLUCC 14-0152 ^T^	Roots of *Dendrobiumharveyanum*	Thailand	MZ463151	MZ463192	NA	NA
*A.descalsii*	AP31118A ^T^	*Ampelodesmosmauritanicus*	Spain	MK014870	MK014837	MK017976	MK017947
*A.dichotomanthi*	LC4950 ^T^	*Dichotomanthestristaniicarpa*	China	KY494697	KY494773	KY705167	KY705096
*A.dicranopteridis*	KUNCC23-14171 ^T^	Living stems of *Dicranopterispedata*	China	OR590342	OR590336	OR634944	OR634950
*A.dongyingensis*	SAUCC 0302 ^T^	Leaves of bamboo	China	OP563375	OP572424	OP573270	OP573264
*A.elliptica*	ZHKUCC 22-0131 ^T^	Dead stems of unknown plant	China	OR164905	OR164952	OR166323	OR166284
*A.endophytica*	ZHKUCC 23-0006 ^T^	Living leaves of *Wurfbainiavillosa*	China	OQ587996	OQ587984	OQ586075	OQ586062
*A.esporiensis*	AP16717	*Phyllostachysaurea*	Spain	MK014878	MK014845	MK017983	MK017954
*A.euphorbiae*	IMI 285638b	*Bambusa* sp.	Bangladesh	AB220241	AB220335	AB220288	NA
*A.fermenti*	KUC21289 ^T^	Seaweeds	South Korea	MF615226	MF615213	MF615231	MH544667
*A.gaoyouensis*	CFCC 52301 ^T^	*Phragmitesaustralis*	China	MH197124	NA	NA	MH236793
*A.gaoyouensis*	CFCC 52302	*Phragmitesaustralis*	China	MH197125	NA	NA	MH236794
*A.garethjonesii*	SICAUCC 22-0027	Bamboo	China	ON228603	ON228659	ON237651	NA
*A.gelatinosa*	GZAAS 20-0107	Bamboo	China	MW481707	MW478889	NA	MW522942
*A.globosa*	KUNCC 23-14210 ^T^	Living stems of *Dicranopterislinearis*	China	OR590347	OR590340	NA	OR634954
*A.gongcheniae*	GDMCC 3.1045^T^	Stems of Oryzameyerianasubsp.granulata	China	PP033259	PP034691	PP033102	PP034683
*A.gongcheniae*	YNE00565	Stems of Oryzameyerianasubsp.granulata	China	PP033260	PP034692	PP033103	PP034684
*A.guangdongensis*	ZHKUCC 23-0004 ^T^	*Wurfbainiavillosa*	China	OQ587994	OQ587982	OQ586073	OQ586060
*A.guizhouensis*	LC5318	Air in karst cave	China	KY494708	KY494784	KY705177	KY705107
*A.hainanensis*	SAUCC 1681 ^T^	Leaves of bamboo	China	OP563373	OP572422	OP573268	OP573262
*A.hispanica*	IMI 326877 ^T^	Beach sands	Spain	AB220242	AB220336	AB220289	NA
*A.hydei*	CBS 114990 ^T^	Culms of *Bambusatuldoides*	China	KF144890	KF144936	KF144982	KF145024
*A.hyphopodii*	SICAUCC 22-0034	Bamboo	China	ON228605	ON228661	ON237653	NA
*A.hysterina*	AP12118	*Phyllostachysaurea*	Spain	MK014877	KM014844	MK017982	MK017953
*A.iberica*	AP10118 ^T^	*Arundodonax*	Portugal	MK014879	MK014846	MK017984	MK017955
*A.intestini*	CBS 135835	Gut of grasshopper	India	KR011352	MH877577	KR011350	KR011351
*A.italica*	AP29118	*Arundodonax*	Italy	MK014881	MK014848	MK017986	NA
*A.jatrophae*	MMI00052 ^T^	Living *Jatrophapodagrica*	India	JQ246355	NA	NA	NA
*A.jiangxiensis*	LC4577 ^T^	*Maesa* sp.	China	KY494693	KY494769	KY705163	KY705092
*A.jiangxiensis*	LC4578	Camelliasinensis	China	KY494694	KY494770	KY705164	KY705093
*A.jinanensis*	SAUCC DL1981 ^T^	Diseased leaves of Bambusaceae sp.	China	OQ592544	OQ615273	OQ613289	OQ613317
*A.kogelbergensis*	CBS 113332	*Cannomoisvirgata*	South Africa	KF144891	KF144937	KF144983	KF145025
*A.koreana*	KUC21332 ^T^	Eggs of *Arctoscopusjaponicus*	South Korea	MH498524	MH498444	MH498482	MH544664
*A.lageniformis*	KUC21686 ^T^	Culms of *Phyllostachysnigra*	South Korea	ON764022	ON787761	ON806636	ON806626
*A.lageniformis*	KUC21687	Culms of *Phyllostachysnigra*	South Korea	ON764023	ON787764	ON806637	ON806627
*A.locuta-pollinis*	LC11683 ^T^	*Brassicacampestris*	China	MF939595	NA	MF939622	MF939616
*A.longistroma*	MFLUCC11-0481 ^T^	Dead culms of bamboo	Thailand	KU940141	KU863129	NA	NA
*A.lophatheri*	CFCC 58975 ^T^	Diseased leaves of *Lophatherumgracile*	China	OR125566	OR133588	OR139980	OR139970
*A.machili*	SAUCC 1175A-4 ^T^	Diseased leaves of *Machilusnanmu*	China	OR739433	OR739574	OR757130	OR753450
*A.machili*	SAUCC 1175	Diseased leaves of *Machilusnanmu*	China	OQ592560	OQ615289	OQ613307	OQ613333
*A.machili*	SAUCC 1176	Diseased leaves of *Machilusnanmu*	China	OQ592559	OQ615288	OQ613306	OQ613332
*A.malaysianum*	CBS 102053 ^T^	*Macarangahullettii*	Malaysia	KF144896	KF144942	KF144988	KF145030
*A.marianiae*	AP18219 ^T^	Dead stems of *Phleumpratense*	Spain	ON692406	ON692422	ON677186	ON677180
*A.marii*	CBS 497.90 ^T^	Beach sands	Spain	AB220252	KF144947	KF144993	KF145035
*A.marina*	KUC21328 ^T^	Seaweeds	South Korea	MH498538	MH498458	MH498496	MH544669
*A.mediterranea*	IMI 326875 ^T^	Air	Spain	AB220243	AB220337	AB220290	NA
*A.minutispora*	1.70E-42 ^T^	Mountain soils	South Korea	LC517882	NA	LC518888	LC518889
*A.montagnei*	AP301120 ^T^	*Arundomicrantha*	Spain	ON692408	ON692424	ON677188	ON677182
*A.mori*	MFLU 18-2514 ^T^	*Morusaustralis*	China	MW114313	MW114393	NA	NA
*A.mukdahanensis*	MFLUCC 22-0056 ^T^	Dead leaves of bamboo	Thailand	OP377735	OP377742	NA	NA
*A.mytilomorpha*	DAOM 214595	Dead blades of *Andropogon* sp.	India	KY494685	NA	NA	NA
*A.neobambusae*	LC7106 ^T^	Leaves of bamboo	China	KY494718	KY494794	KY705186	KY806204
*A.neochinense*	CFCC 53036 ^T^	*Fargesiaqinlingensis*	China	MK819291	NA	MK818547	MK818545
*A.neosubglobosa*	JHB 007 ^T^	Bamboo	China	KY356090	KY356095	NA	NA
*A.obovata*	LC4940 ^T^	*Lithocarpus* sp.	China	KY494696	KY494772	KY705166	KY705095
*A.obovata*	LC8177	*Lithocarpus* sp.	China	KY494757	KY494833	KY705225	KY705153
*A.oenotherae*	CFCC 58972	Diseased leaves of *Oenotherabiennis*	China	OR125568	OR133590	OR139982	OR139972
*A.olivata*	CGMCC 3.25514 ^T^	soil	China	OR680531	OR680598	OR843234	OR858925
*A.olivata*	ZY 22.053	soil	China	OR680532	OR680599	OR843235	OR858926
*A.ovata*	CBS 115042 ^T^	*Arundinariahindsii*	China	KF144903	KF144950	KF144995	KF145037
*A.pallidesporae*	ZHKUCC 22-0129 ^T^	Dead wood of unknown host	China	OR164903	OR164950	NA	NA
*A.paraphaeosperma*	KUC21488	Culms of bamboo	South Korea	ON764024	ON787763	ON806638	ON806628
*A.phragmitis*	CPC 18900 ^T^	*Phragmitesaustralis*	Italy	KF144909	KF144956	KF145001	KF145043
*A.phyllostachydis*	MFLUCC 18-1101 ^T^	*Phyllostachysheteroclada*	China	MK351842	MH368077	MK291949	MK340918
*A.piptatheri*	SAUCC BW0455	Diseased leaves of *Indocalamuslongiauritus*	China	OR739430	OR739571	OR757127	OR753447
*A.pseudohyphopodii*	KUC21680 ^T^	Culms of *Phyllostachyspubescens*	South Korea	ON764026	ON787765	ON806640	ON806630
*A.pseudomarii*	GUCC 10228 ^T^	Leaves of *Aristolochiadebilis*	China	MT040124	NA	MT040166	MT040145
*A.pseudoparenchymatica*	LC7234 ^T^	Leaves of bamboo	China	KY494743	KY494819	KY705211	KY705139
*A.pseudorasikravindrae*	KUMCC 20-0208 ^T^	*Bambusadolichoclada*	China	MT946344	NA	MT947367	MT947361
*A.pseudosinensis*	SAUCC 0221	Leaves of bamboo	China	OP563377	OP572426	OP573272	OP573266
*A.pseudospegazzinii*	CBS 102052 ^T^	*Macarangahullettii*	Malaysia	KF144911	KF144958	KF145002	KF145045
*A.pterosperma*	CPC 20193 ^T^	*Lepidospermagladiatum*	Australia	KF144913	KF144960	KF145004	KF145046
*A.pusillisperma*	KUC21321 ^T^	Seaweeds	South Korea	MH498533	MH498453	MH498491	MN868930
***A.qiannanensis***	**RCEF7610**	**Diseased culms of bamboo**	**China**	**PQ526600**	**PQ530550**	**PQ538539**	**PQ538535**
***A.qiannanensis***	**RCEF7611** ^T^	**Diseased culms of bamboo**	**China**	**PQ526599**	**PQ530549**	**PQ538538**	**PQ538536**
*A.qinlingensis*	CFCC 52303 ^T^	*Fargesiaqinlingensis*	China	MH197120	NA	NA	MH236795
*A.rasikravindrae*	LC8179	Brassica rapa	China	KY494759	KY494835	KY705227	KY705155
*A.sacchari*	CBS 372.67	Air	Not mentioned	KF144918	KF144964	KF145007	KF145049
*A.saccharicola*	CBS 191.73	Air	Netherlands	KF144920	KF144966	KF145009	KF145051
*A.sargassi*	KUC21232	Seaweeds	South Korea	KT207750	NA	KT207648	MH544676
*A.sasae*	CPC 38165 ^T^	Dead culms of *Sasaveitchii*	Netherlands	MW883402	MW883797	MW890120	MW890104
*A.septata*	GZCC 20-0109	Bamboo Food	China	MW481712	MW478891	MW522961	MW522944
*A.serenensis*	IMI 326869 ^T^	Excipients, atmosphere and home dust	Spain	AB220250	AB220344	AB220297	NA
*A.setariae*	CFCC 54041 ^T^	Decaying culms of *Setariaviridis*	China	MT492004	NA	MT497466	MW118456
*A.setostroma*	KUMCC 19-0217	Dead branches of bamboo	China	MN528012	MN528011	NA	MN527357
*A.sichuanensis*	HKAS 107008 ^T^	Dead culms of Poaceae	China	MW240648	MW240578	MW775605	MW759536
*Apiospora* sp.	SAUCC 1429	NA	China	OQ592558	OQ615287	OQ613305	OQ613331
*Apiospora* sp.	SAUCC 1430	NA	China	OQ592557	OQ615286	OQ613304	OQ613330
*A.sphaerosperma*	CBS 114315	Leaves of *Hordeumvulgare*	Iran	KF144905	KF144952	KF144997	KF145039
*A.stipae*	CPC 38101 ^T^	Dead culms of *Stipagigantea*	Spain	MW883403	MW883798	MW890121	MW890082
*A.subglobosa*	MFLUCC 11-0397 ^T^	Dead culms of bamboo	Thailand	KR069112	KR069113	NA	NA
*A.subrosea*	LC7291	Leaves of bamboo	China	KY494751	KY494827	KY705219	KY705147
*A.taeanensis*	KUC21359	Seaweeds	South Korea	MH498513	NA	MH498471	MN868935
*A.thailandica*	MFLUCC 15-0202 ^T^	Dead culms of bamboo	Thailand	KU940145	KU863133	NA	NA
*A.tropica*	MFLUCC 21-0056	Dead culms of Bambusoideae	Thailand	OK491657	OK491653	NA	NA
*A.vietnamensis*	IMI 99670 ^T^	*Citrussinensis*	Vietnam	KX986096	KX986111	KY019466	NA
*A.wurfbainiae*	ZHKUCC 23-0009	*Wurfbainiavillosa*	China	OQ587999	OQ587987	OQ586078	OQ586065
*A.xenocordella*	CBS 478.86 ^T^	Soils from roadway	Zimbabwe	KF144925	KF144970	KF145013	KF145055
***A.xiangxiense***	**RCEF20001** ^T^	**Diseased culms of bamboo**	**China**	**OR687308**	**PQ530553**	**OR712910**	**OR712909**
***A.xiangxiense***	**RCEF20002**	**Diseased culms of bamboo**	**China**	**OR687307**	**PQ530548**	**OR712908**	**OR712907**
*A.xishuangbannaensis*	KUMCC 21-0696	*Rhinolophuspusillus*	China	ON426833	OP363249	OR025931	OR025970
*A.yunnana*	DDQ 00281	Phyllostachysnigra	China	KU940148	KU863136	NA	NA
*A.yunnanensis*	ZHKUCC 23-0014 ^T^	Dead stems of grass	China	OQ588004	OQ587992	OQ586083	OQ586070
*Arthriniumcaricicola*	AP23518	*Carexericetorum*	China	MK014871	MK014838	MK017977	MK017948
*Arthriniumcaricicola*	CBS 145903	Dead and attached leaves	Germany	MN313782	MN317266	MN313861	NA

The DNA sequences were aligned using the MAFFT v7 with the G-INS-I option (Katoh et al. 2019). Sequences were manually edited as necessary using BioEdit v7.1.9 (Hall 1999). The combined loci were analyzed using maximum likelihood (ML) and Bayesian inference (BI) methods. Combined sequences of ITS-LSU-*TUB2*-*TEF1* were performed in SequenceMatrix v1.7.8 (Vaidya et al. 2011). The ML analysis was conducted using the TNe+I+R4 model and 1000 bootstrap replicates. The ML analysis was designed with IQ-TREE (Trifinopoulos et al. 2016). In Bayesian inference analysis, the best-fit substitution models for different datasets were estimated using MrModeltest v2.3 based on the implementation of the Akaike information criterion (AIC) (Nylander 2004). Posterior probabilities (PP) were determined by Markov Chain Monte Carlo sampling (MCMC) under the estimated model of evolution (Zhaxybayeva and Gogarten 2002). Four simultaneous Markov chains were run for 20 million generations, and trees were sampled every 1000 generations. The run was stopped automatically when the average standard deviation of split frequencies fell below 0.01. The first 25% trees, which represented the burn-in phase of the analyses, were discarded, and the remaining trees were used for calculating PP in the majority rule consensus tree. Phylogenetic trees were subsequently visualized and refined using the Interactive Tree of Life (iTOL) online platform (Letunic and Bork 2019).

### ﻿Pathogenicity assay

Fresh bamboo samples were collected from the campus of Anhui Agricultural University in Anhui Province, China, to validate Koch’s postulates. Bamboo culms were cut into 30 cm sections, sterilized with 75% ethanol spray, and wounded using sterile drills. Mycelial plugs (5 mm in diameter) from the edge of each isolate colony were placed onto the artificial wounds, while control pieces received PDA plugs without fungal inoculum (Zheng et al. 2022). All treated culms were placed in moisture chambers with sterile wet cotton to maintain humidity and incubated at 25 °C under a 12-hour light/12-hour dark cycle. Symptom development was observed daily, and each treatment was replicated six times.

## ﻿Results

### ﻿Disease symptoms and isolation of the pathogen

**Figure 1.**
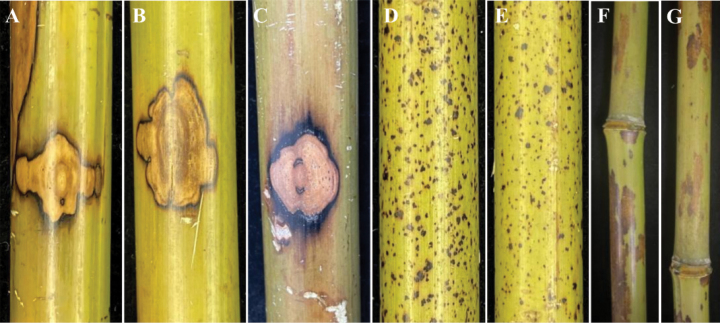
Symptoms of disease in naturally infected bamboo in the field.

From the field survey, disease symptoms developed on the culm of the bamboo. The typical symptoms: (I) started with brown spots and irregular shapes that gradually enlarged, with dark edges and spread around, sometimes forming symmetrical or lobed patterns (Fig. 1A–C). (II) started with black spots and irregular shapes; each spot remained relatively small and did not expand significantly. In the later stages, they densely covered bamboo culm and exhibited chlorosis in the bamboo culms (Fig. 1D, E). (III) Irregular brownish lesions spread extensively, sometimes coalescing into large patches, covering a significant area of the bamboo culm (Fig. 1F, G).

A total of 37 isolates were obtained on PDA. As the colony morphology of the isolates was uniform, two representative isolates from each group were selected for further analysis: (I). RCEF20001 and RCEF20002; (II). RCEF20000 and RCEF20003; (III). RCEF7610 and RCEF7611.

### ﻿Phylogenetic analysis

A comprehensive dataset integrating ITS, LSU, *TUB2*, and *TEF1* sequences was constructed from 131 strains, including six newly sequenced isolates, with *Arthriniumcaricicola* (CBS 145903 and AP23518) designated as the outgroup. Multi-locus sequences contained 2,544 characters, including gaps with ITS (1-433), LSU (434-1229), *TUB2* (1230-1678), and *TEF1* (1679-2544).

**Figure 2.**
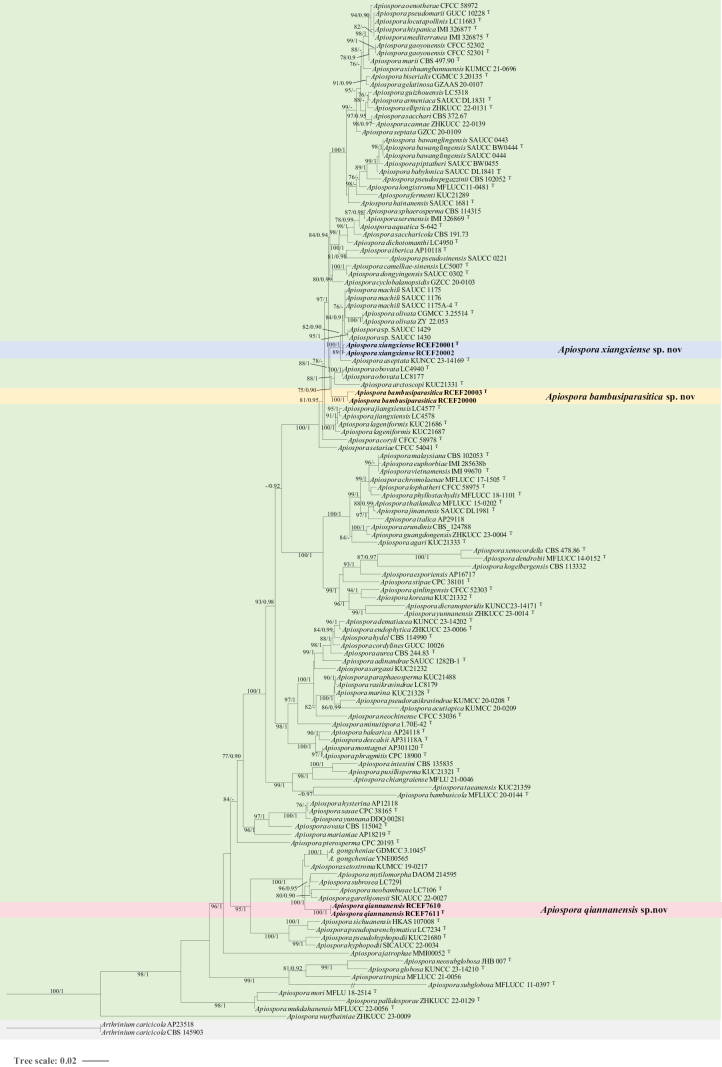
Phylogenetic tree of *Apiospora* based on a concatenated data matrix of ITS, LSU, *TUB2*, and *TEF1*. Bootstrap support values (> 75%) and posterior probabilities (> 0.9) are given at the nodes (ML/PP). The tree is rooted with *Arthriniumcaricicola* CBS 145903 and AP23518. The novel species were highlighted. “T” indicates a type culture.

The phylogenetic trees derived from ML and BI analyses exhibited consistent topologies, with the ML tree, including MLBP and BIPP values, depicted in Fig. 2. Phylogenetic analysis revealed that the six strains represented three new species lineages, which are now recognized as *A.bambusiparasitica*, *A.qiannanensis*, and *A.xiangxiense*.

### ﻿Taxonomy

#### ﻿ Apiospora bambusiparasitica

Taxon classificationFungiAmphisphaerialesApiosporaceae

X.Y. Chang & M.J. Chen sp. nov.

D701F331-322C-5FB4-BE09-1C8DCE26A83B

851766

Fig. 3

##### Etymology.

The name refers to the species that is capable of infecting the culm of bamboo.

##### Typification.

China • Hunan Province, Xiangxi Tujia and Miao Autonomous Prefecture, Ningyuan County, Jiuyi Mountain (25°24'N, 111°58'E), on diseased culms of bamboo, November 2022; X.H. Yue, holotype H5, ex-type RCEF20003.

**Figure 3.**
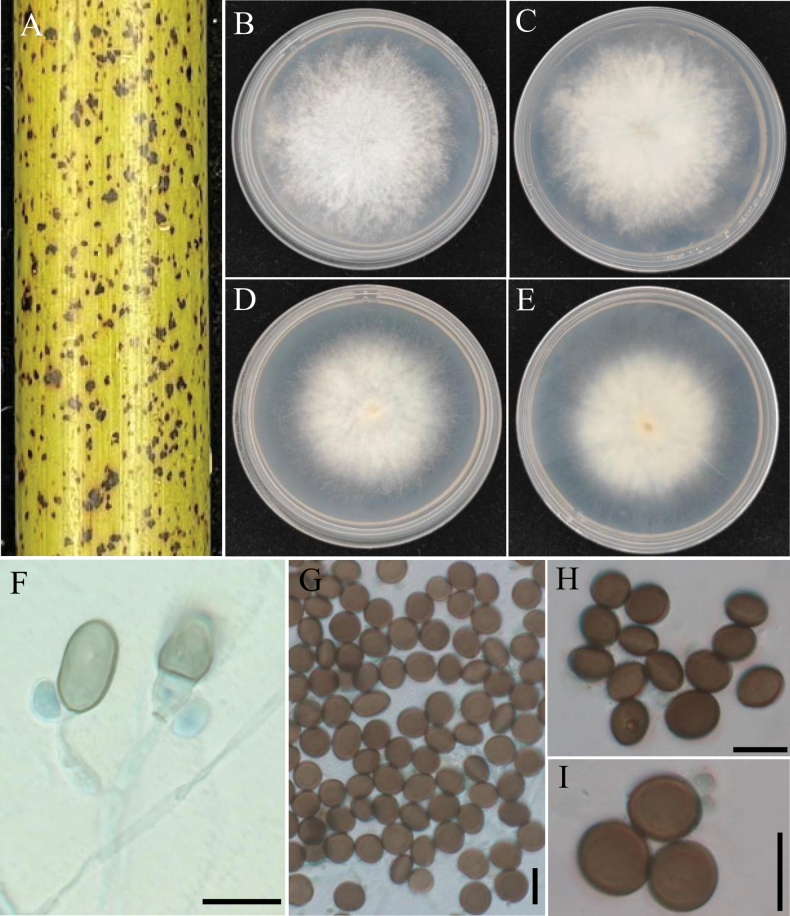
*Apiosporabambusiparasitica* (from ex-type living cultures RCEF20003) **A** diseased culms of bamboo **B, C** upper view and reverse view of culture on PDA**D, E** upper view and reverse view of culture on MEA **F** conidiogenous cells giving rise to conidia **G–I** conidia with pale germ slit. Scale bars: 10 µm.

##### Description.

***Asexual morph***: Hyphae 1.5–5.0 µm diam, hyaline, branched, septate. ***Conidiogenous cells*** hyaline to pale brown, smooth, erect or flexuous, scattered or aggregated in clusters on hyphae, ampulliform to clavate, 7.0–17.0 × 2.0–4.5 µm (*x̄* = 9.6 ± 2.6 × 2.7 ± 0.7, n = 40), apical neck 6.0–10.0 µm long, basal part 3.0–6.0 µm long. ***Conidia*** 7.0–11.5 × 6.0–10.5 µm (*x̄* = 9.2 ± 0.9 × 8.1 ± 1.1, n = 40), brown, smooth to finely roughened, granular, globose to ellipsoid in surface view, usually with a longitudinal, hyaline, germ-slit. ***Sexual morph***: Undetermined.

##### Culture characteristics.

Colonies on PDA fluffy, spreading, margin irregular, with abundant aerial mycelia, surface and reverse white to grey, reaching 9 cm in 8 d at 25 °C. On MEA, the colony is thick in the middle and thin at the edges. The margin is irregular, the surface white, and the central color on the colony’s reverse side is characterized by a deeper, brownish-yellow tone that extends towards the periphery and transitions to a lighter, pale yellow shade.

##### Additional specimens examined.

China • Hunan Province, Ningyuan County, diseased on culms of bamboo, November 2022, other living culture RCEF20000.

##### Note.

Phylogenetic analyses confirmed that *A.bambusiparasitica* formed an independent clade (1.0 BIPP and 100% MLBS), exhibiting a close evolutionary relationship with *A.arctoscopi* and *A.obovata*. Based on a BLASTN search of the GenBank database, it was found that *A.bambusiparasitica* shares high similarities with the following strains: *A.arctoscopi* strain KUC21331 (86.48% in ITS, 98.9% in LSU, 92.2% in *TEF1*, 92.91% in *TUB2*); *A.obovata* strain LC4940 (90.03% in ITS, 95.77% in LSU, 93.42% in *TEF1*, 95.27% in *TUB2*); strain LC8177 (90.15% in ITS, 95.77% in LSU, 93.42% in *TEF1*, 95.27% in *TUB2*).

Morphologically, *A.bambusiparasitica* and *A.obovata* show distinct differences. *Apiosporaobovata* forms darker colonies and produces significantly longer, ellipsoidal conidia, measuring 16.0–31.0 × 9.0–16.0 µm, whereas *A.bambusiparasitica* has spherical to oval conidia, measuring 8.6–15.4 × 6.7–10.2 µm. *Apiosporabambusiparasitica* and *A.arctoscopi* are morphologically similar, with conidia of comparable size and overlapping dimensions. However, *A.arctoscopi* forms thicker colonies with more developed hyphae. Additionally, the two species exhibit significant ecological differences in host association, as *A.arctoscopi* is associated with *Arctoscopusjaponicus*, while *A.bambusiparasitica* is associated with bamboo. Current fungal taxonomy emphasizes the importance of host association. For details, see Table 2. Thus, both morphological and molecular evidence support *A.bambusiparasitica* as a new species.

**Table 2.** Synopsis of morphological characteristics of *A.bambusiparasitica* and its closely related species.

Species	Isolation source	Country	Colony morphology (on PDA)	Conidia		References
				Shape	Diam (μm)	
*A.obovata*	*Lithocarpus* sp.	China	White to olivaceous-grey; Reaching 9 cm in 7 days	a. Roughened, globose to subglobose; b. obovoid, occasionally elongated to ellipsoidal.	a. 11.0–16.5; b. 16.0–31.0 × 9.0–16.0	Wang et al. (2018)
*A.arctoscopi*	Egg masses of *Arctoscopusjaponicus*	Korea	Creamy white;5-7 cm in 5 days	globose to elongate ellipsoid	9.5–13 × 7.5–12	Kwon et al. (2021)
*A.bambusiparasitica*	Diseased culms of Bamboo	China	White to grey; Reaching 9 cm in 8 days	globose to elongate ellipsoid	8.6–15.4 × 6.7–10.2	**This study**

#### ﻿ Apiospora qiannanensis

Taxon classificationFungiAmphisphaerialesApiosporaceae

X.Y. Chang & M.J. Chen sp. nov.

FF96AF42-325C-52F1-A5AB-106E287BA450

856457

Fig. 4

##### Etymology.

The name refers to the locality where the type specimens were collected, Qiannan Buyi and Miao Autonomous Prefecture, Guizhou Province, China.

**Figure 4.**
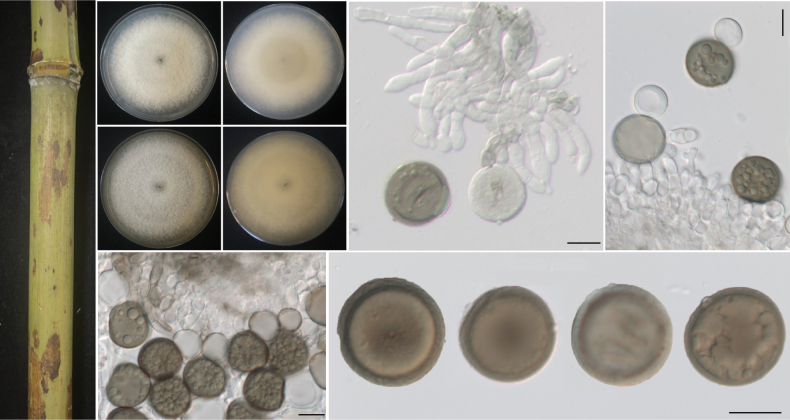
*Apiosporaqiannanensis* (from ex-holotype strain RCEF7610) **A** diseased culms of bamboo **B, C** upper view and reverse view of culture on PDA**D, E** upper view and reverse view of culture on MEA **F–H** conidiogenous cells giving rise to conidia **I** conidia. Scale bars: 10 μm.

##### Typification.

China • Guizhou Province, Qiannan Buyi and Miao Autonomous Prefecture, Libo County (25°25'N, 107°53'E), on diseased culms of bamboo, May. 2023, X.H. Yue, holotype GZ15, ex-type RCEF7610.

##### Description.

***Asexual morph***: Hyphae 1.5–6.0 µm diam, hyaline to pale brown, branched, septate. ***Conidiophores*** hyaline to pale brown, smooth, erect or ascending, simple, flexuous, subcylindrical, and grouped together. ***Conidiophores*** aggregated in brown sporodochia, smooth, hyaline to brown, up to 30 µm long, 3.0–4.0 µm width. ***Conidiogenous cells*** 9.5–23.0 × 3.0–5.5 µm (*x̄* = 15.0 ± 4.50 × 4.3 ± 0.9, n = 40), pale brown, smooth, doliiform to subcylindrical. ***Conidia*** 16.5–20.8 µm (*x̄* = 18.5 μm, n = 40), pale brown to dark brown, smooth, globose to subglobose. ***Sexual morph***: Undetermined.

##### Culture characteristics.

Colonies on PDA are fluffy, spreading, and circular, with moderate aerial mycelia, flocculent cotton, surface, and reverse white to grey, reaching 60 mm in 7 d at 25 °C. On MEA, surface grey-white with abundant mycelia, reverse greyish without patches.

##### Additional specimens examined.

China • Hunan Province, Ningyuan County, diseased on culms of bamboo, May 2023, other living culture RCEF7611.

##### Note.

Phylogenetic analyses confirmed that *A.qiannanensis* formed an independent clade (1.0 BIPP and 100% MLBS), exhibiting a close evolutionary relationship with *A.setostroma*, *A.mytilomorpha*, *A.subrosea*, *A.neobambusae*, and *A.garethjonesii*. Based on a BLASTN search of the GenBank database, it was found that *A.qiannanensis* exhibits some differences in the ITS, LSU, *TUB2*, and *TEF1* sequences compared to closely related species: *A.setostroma* strain KUMCC 19-0217 (92.65% in ITS, 99.16% in LSU, 95.01% in *TEF1*); *A.mytilomorpha* strain DAOM 2145955 (96.28% in ITS); *A.subrosea* strain LC7291 (90.33% in ITS, 99.02% in LSU, 94.38% in *TEF1*, 99.25% in *TUB2*); *A.neobambusae* strain LC7106 (89.16% in ITS, 99.16% in LSU, 95.22% in *TEF1*, 91.94% in *TUB2*); *A.garethjonesii* strain SICAUCC 22-0027 (93.65% in ITS, 99.29% in LSU, 94.50% in *TUB2*); *A.gongcheniae* strain GDMCC 3.1045 (95.44% in ITS, 99.41% in LSU, 93.14% in *TEF1*, 91.77% in *TUB2*).

Morphologically, colony characteristics of *A.mytilomorpha* are lacking, and the asexual morphology of *A.garethjonesii* has not been described. We compared the existing morphological data and found that these closely related species have certain differences. *A.setostroma* and *A.subrosea* produce pigments in the later stages of colonies, while the others do not. *Apiosporaqiannanensis*, *A.mytilomorpha*, and *A.neobambusae* differ in conidia shape (globose to subglobose vs. fusiform or boat-shaped vs. subglobose to ellipsoid) and size (16.5–20.8 μm vs. 20–30 × 6–8.5 μm vs. 11.5–15.5 × 7.0–14.0 μm). In addition, *A.qiannanensis* differs from *A.gongcheniae* in having larger conidia (16.5–20.8 µm) compared to A.gongcheniae (8.0–17.0 × 6.8–16.1 µm). Although some morphological features overlap among these taxa, significant genetic divergence is evident, underscoring their distinct species boundaries. For details, see Table 3. Based on molecular and morphological evidence, we propose *A.qiannanensis* as a new species.

**Table 3.** Synopsis of morphological characteristics of *A.qiannanensis* and its closely related species.

Species	Isolation source	Country	Colony morphology (on PDA)	Conidia		References
				Shape	Diam (μm)	
*A.qiannanensis*	Diseased culms of bamboo	China	White to grey; Reaching 60 mm in 7 days	Globose to subglobose	16.5–20.8	**This study**
*A.gongcheniae*	Stems of Oryzameyerianasubsp.granulata	China	Greyish, reverse light orange; Reaching 90 mm in 7 days	Globose to subglobose	8.0–17.0 × 6.8–16.1	Yan and Zhang (2024)
*A.setostroma*	Dead branches of bamboo	China	Initially white, becoming greyish, reverse reddish; Reaching 35 mm in 7 days	Subglobose to obovoid, 0–1-septate	18–20 × 15–19	Jiang et al. (2019)
*A.mytilomorpha*	Dead blades of Andropogon	India	Undetermined	Fusiform or boat-shaped	20-30 × 6-8.5	Bhat and Kendrick (1993)
*A.subrosea*	Bamboo	China	Initially white, becoming light pink on surface, reverse peach-puff; Reaching 10 cm in 8 days	Globose to subglobose or ellipsoidal	12.0–17.5 × 9.0–16.0	Wang et al. (2018)
*A.neobambusae*	Leaf of bamboo	China	White to grey	Subglobose to ellipsoid	11.5–15.5 × 7.0–14.0	Wang et al. (2018)
*A.garethjonesii*	Dead culms of bamboo	China	White; Reaching 40 cm in 7 days	Undetermined	Undetermined	Dai et al. (2016)

#### ﻿ Apiospora xiangxiense

Taxon classificationFungiAmphisphaerialesApiosporaceae

X.Y. Chang & M.J. Chen sp. nov.

03AB0B17-CACF-5B6B-8AC6-58393F093276

851765

Fig. 5

##### Etymology.

The name refers to the locality where the type specimens were collected, Xiangxi Tujia and Miao Autonomous Prefecture, Hunan Province, China.

**Figure 5.**
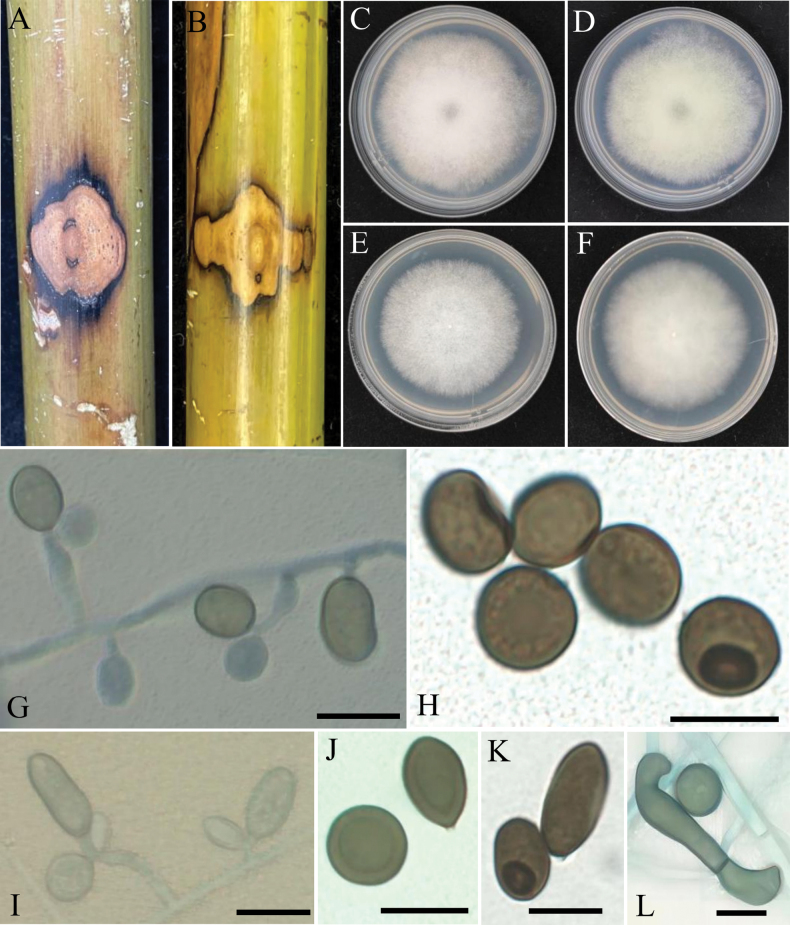
*Apiosporaxiangxiense* (from ex-type living cultures RCEF20001) **A, B** diseased culms of bamboo **C, D** upper view and reverse view of culture on PDA**E, F** upper view and reverse view of culture on MEA **G, H** conidiogenous cells giving rise to conidia **I–K** conidia **L** sterile cells and conidia. Scale bars: 10 µm.

##### Typification.

China • Hunan Province, Xiangxi Tujia and Miao Autonomous Prefecture, Ningyuan County, Jiuyi Mountain (25°24'N, 111°58'E), on diseased culms of bamboo, November 2022, X.H. Yue, holotype H2 (stored in a metabolically inactive state), ex-type living cultures RCEF20001.

##### Description.

***Asexual morph***: Hyphae 1.5–5.0 µm diam, hyaline, branched, septate. ***Conidiogenous cells*** 2.0–15.5 × 1.4–3.9 µm (*x̄* = 8.1 ± 3.9 × 2.4 ± 0.7, n = 40), aggregated in clusters on hyphae or solitary, at first hyaline, becoming pale brown, basauxic, polyblastic, sympodial, erect, cylindrical. ***Conidia*** 8.6–15.4 × 6.7–10.2 µm (*x̄* = 10.3 ± 1.5 × 8.3 ± 1.0, n = 40), brown, smooth to granular, globose to elongate ellipsoid in surface view, lenticular in side view, pale equatorial slit, with a central scar, 3.5 to 5.5 µm diam. Sterile cells forming on solitary loci on hyphae, brown, finely roughened, subcylindrical to clavate. ***Sexual morph***: Undetermined.

##### Culture characteristics.

Colonies on PDA are fluffy, spreading, circular, with abundant aerial mycelia, surface and reverse white to grey, sometimes with pale yellow, reaching 9 cm in 8 d at 25 °C. On MEA, slower growth, surface white, reverse white, and slightly yellowish.

##### Additional specimens examined.

China • Hunan Province, Ningyuan County, diseased on culms of bamboo, November 2022, other living culture RCEF20002.

##### Note.

Phylogenetic analyses confirmed that *A.xiangxiense* formed an independent clade, exhibiting a close evolutionary relationship with *A.aseptata*, *A.olivata*, and *A.machili* (1.0 BIPP and 100% MLBS).

However, *A.xiangxiense* differs from *A.aseptata* in several key aspects, including conidial size (8.6–15.4 × 6.7–10.2 µm vs. 7–9.5 (–13) µm). Based on nucleotide comparisons, *A.xiangxiense* differs from *A.aseptata* by 0.69% in ITS, 0.16% in LSU, 2.36% in *TUB2*, and 0.49% in *TEF1*. *Apiosporaxiangxiense* also differs from *A.machili* by having longer conidia (8.6–15.4 × 6.7–10.2 µm vs. 7.1–9.5 × 5.6–8.8 µm) and more elongated conidiogenous cells (2.0–15.5 × 1.4–3.9 µm vs. 6.0–8.0 × 2.5–4.0 µm). *Apiosporaxiangxiense* differs from *A.olivata* by having longer conidia (8.6–15.4 × 6.7–10.2 µm) compared to *A.olivata* (8–12 × 5.5–8 µm), with sequence differences of 7.52% in ITS, 1.22% in LSU, and 1.94% in *TUB2*. Furthermore, their isolation sources are different.

For details, see Table 4. Thus, both morphological and molecular evidence support *A.xiangxiense* as a new species.

**Table 4.** Synopsis of morphological characteristics of *A.xiangxiense* and its closely related species.

Species	Isolation source	Country	Colony morphology (on PDA)	Conidia		References
				Shape	Diam (μm)	
*A.aseptata*	Healthy leaf of *Dicranopterispedata*	China	Grey-brown; 5 cm in 10 days	Globose or sub globose	7–9.5 (–13)	Zhang et al. (2023)
*A.machili*	Diseased leaves of *Machilusnanmu*	China	Ivory; 69.7–78.8 mm cm in 7 days	Globose to subglobose	7.1–9.5 × 5.6–8.8	Liu et al. (2024)
*A.olivata*	Green belt soil	China	initially white, becoming curry on the surface; reverse pale green; more than 90 mm in 14 days	a. olivary; b. subglobose to globose	a. 8–12 × 5.5–8 μm; b. 8–11.5 μm	Zhang et al. (2024)
*A.xiangxiense*	Diseased culms of Bamboo	China	white to grey, sometimes with pale yellow; Reaching 9 cm in 8 days	globose to elongate ellipsoid	8.6–15.4 × 6.7–10.2	**This study**

### ﻿Pathogenicity tests

**Figure 6.**
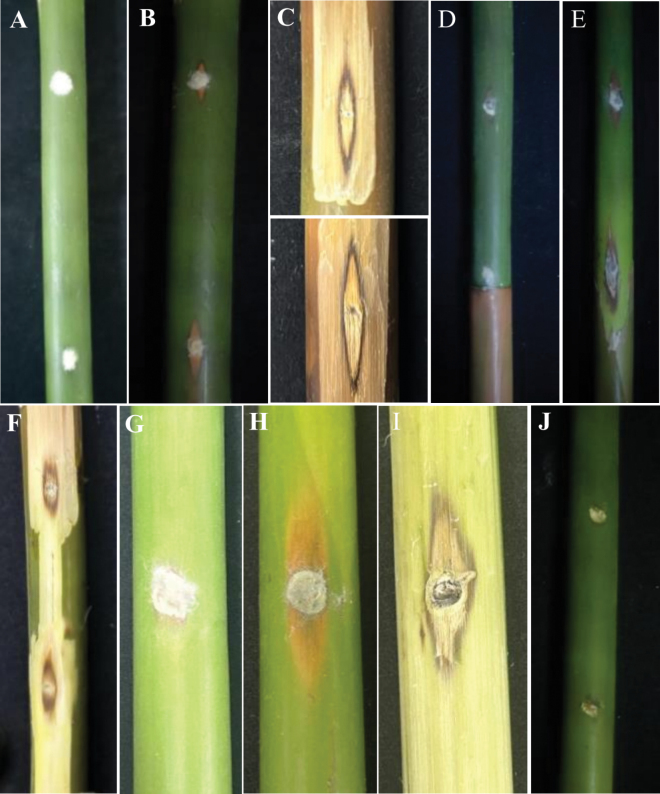
Pathogenicity test **A** symptoms on bamboo culm inoculated with the isolate *A.xiangxiense* RCEF20001 after 3 days **B** inoculation with RCEF20001 strain after 5 days **C** details under the diseased tissues **D** symptoms on bamboo culm inoculated with the isolate *A.bambusiparasitica* RCEF20000 after 3 days **E** inoculation with RCEF20000 strain after 5 days **F** details under the diseased tissues **G** symptoms on bamboo culm inoculated with the isolate *A.qiannanensis* RCEF7611 after 3 days **H** inoculation with RCEF7611 strain after 5 days **I** details under the diseased tissues **J** bamboo inoculated with PDA plug.

To determine the pathogenicity of the three new species isolates, three representative strains (RCEF20001, RCEF20000, and RCEF7611) were selected and inoculated onto fresh bamboo culms using a wound inoculation method. All three isolates were able to induce necrotic lesions. Inoculation with *A.xiangxiense* RCEF20001 resulted in the formation of gray-brown diamond-shaped lesions at the wound site after three days. Upon removing the epidermis, the internal lesions exhibited regular hollow black-brown diamond-shaped spots, which were larger than those observed on the surface (Fig. 6A–C). Inoculation with *A.bambusiparasitica* RCEF20000 caused rotting, diamond-shaped lesions at the wound site, with internal lesions displaying elliptical to scattered black-brown spots after the epidermis was scraped off (Fig. 6D–F). Inoculation with *A.qiannanensis* RCEF7611 resulted in gray-brown oval to diamond-shaped lesions at the wound site after three days. Scraping off the epidermis revealed hollow black-brown diamond-shaped spots, which were smaller than those seen on the surface (Fig. 6H, I). The control group was subjected to the same wound treatment as the experimental groups, but without pathogen inoculation, and no visible symptoms were observed in the control group (Fig. 6J). The symptoms observed were similar to those of naturally infected bamboo. Furthermore, the same fungus was consistently recovered from the experimentally inoculated bamboo. Deposits of the isolates are maintained at the Research Center for Entomogenous Fungi (RCEF), Anhui Agricultural University, Anhui Province, China.

## ﻿Discussion

In this study, 37 isolates of *Apiospora* (Apiosporaceae, Amphisphaeriales, Sordariomycetes) were obtained from diseased culms of bamboo in China (Hunan and Guizhou Provinces). Based on morphological and culture characteristics and phylogenetic analyses of combined ITS, LSU, *TUB2*, and *TEF1* sequence data, three novel species were identified, namely *Apiosporabambusiparasitica*, *A.xiangxiense*, and *A.qiannanensis*. These findings were confirmed through both morphological and molecular characterization, verifying the taxonomic classification of the three species.

*Apiospora* is a cosmopolitan genus distributed across tropical, subtropical, and temperate climates, primarily associated with Poaceae, but also known to colonize a wide range of other hosts (Pintos and Alvarado 2021; Yan and Zhang 2024; Zhang et al. 2024). The strains analyzed in this study were isolated from bamboo in the subtropical regions of China (Guizhou and Hunan), further validating the previously described ecological characteristics of the genus.

According to data from Index Fungorum (accessed on October 28, 2024), the genus *Apiospora* has been recognized to have 196 species. Among them, many species of *Apiospora* are known to be associated with various living and decaying plant materials, and several *Apiospora* species act as plant pathogens. Such as *A.marii*, which causes olive tree dieback in Italy (Gerin et al. 2020); *A.phaeospermum*, which causes leaf necrosis in the olive crop in Sicily (Lo Piccolo et al. 2014); *A.arundinis*, which causes kernel blight of barley in the USA, *Phyllostachyspraecox* brown culm streak disease in Nanjing, leaf blight on tea plants in China, leaf edge spot of peach in China, and culm rhomboid rot of Moso Bamboo (Martínez-Cano et al. 1992; Chen et al. 2014; Thangaraj et al. 2019; Ji et al. 2020; Zheng et al. 2022). The three species in this study were isolated from diseased culms of bamboo, and we verified their pathogenicity to bamboo under laboratory conditions. In the field, the disease symptoms caused by these fungi typically manifest as brown or black lesions of irregular shape on bamboo culms. These lesions may expand, coalesce, and in some cases, lead to extensive necrotic patches, resembling what we refer to as bamboo culm piebald-spot disease. Our pathogenicity tests confirmed that the three newly described species can induce similar symptoms under laboratory conditions, providing a theoretical basis for future research on bamboo disease management and control strategies.

In terms of biological applications, numerous *Apiospora* species produce bioactive secondary metabolites, potentially offering a promising source for pharmacological and medicinal research. For instance, *Apiospora* has shown strong antifungal activity against various plant pathogens (Hong et al. 2015). *A.saccharicola*, isolated from *Miscanthus* sp., is known to produce enzymes of industrial significance (Shrestha et al. 2015). *A.rasikravindrae*, isolated from *Coleusamboinicus*, exhibits notable cytotoxicity against WiDr cells and displays effective antibacterial activity against *Staphylococcusaureus* and *Escherichiacoli* (Astuti et al. 2021). Metabolites from *A.arundinis*, isolated from *Aconitumbrevicalcaratum*, show cytotoxic effects on breast cancer cell lines (Shu et al. 2022), while *A.arundinis* MA30, derived from sea anemones, demonstrates significant anti-inflammatory activity (Lee et al. 2024). Whole-genome sequencing with antiSMASH analysis identified six and ten NR-PKS gene clusters in *A.malaysianum* and *A.koreana*, respectively, which may encode known or novel quinone compounds with notable biological functions (Christiansen et al. 2021). *Apiospora* holds substantial potential for synthesizing diverse secondary metabolites. However, many novel species, including new species in this study, remain underexplored. Future research necessitates further exploration of the biological applications of both known and newly discovered *Apiospora* species to comprehensively elucidate their biological properties.

In conclusion, this study provides a detailed account of three new species of *Apiospora* from China and emphasizes the importance of integrating morphological and molecular data for accurate species identification. Given their potential ecological and economic impacts on bamboo, further research is warranted. Comprehensive taxonomic and ecological investigations will offer valuable insights for potential biotechnological applications and enhance our understanding of this genus and its broader ecological and medicinal significance.

## Supplementary Material

XML Treatment for Apiospora bambusiparasitica

XML Treatment for Apiospora qiannanensis

XML Treatment for Apiospora xiangxiense

## ﻿Additional information

### Author ORCIDs

Xiaoyun Chang https://orcid.org/0000-0002-0093-9582

Yuanyuan Wang https://orcid.org/0009-0007-1801-3984

Tao Xu https://orcid.org/0009-0009-0028-6508

Guangshuo Li https://orcid.org/0000-0002-7285-0712

Xianghua Yue https://orcid.org/0000-0003-2165-7653

Mingjun Chen https://orcid.org/0000-0002-1439-7796

### Data availability

All of the data that support the findings of this study are available in the main text.
